# Evaluation of polymethyl-methacrylate and acetal denture base resins processed by two different techniques before and after nano-chlorohexidine surface treatment

**DOI:** 10.1186/s12903-023-03718-0

**Published:** 2023-12-08

**Authors:** Salma M. Fathy, Mahmoud Saad Abdel-Halim, Samy El-Safty, Amira M. El-Ganiny

**Affiliations:** 1 Dental Biomaterials Department, Faculty of Oral and Dental Medicine, Zagazig University, Zagazig, Egypt, and Faculty of Dentistry, Badr University, Cairo, Egypt; 2 Microbiology and Immunology Department, Faculty of Pharmacy, Zagazig University, Zagazig, Egypt; 3 Dental Biomaterials Department, Faculty of Dentistry, Tanta University, Tanta, Egypt

**Keywords:** Antimicrobial efficacy, Biofilm, Flexible denture bases, Self-cleaning dental materials, Surface roughness, Thermoplastic denture resin

## Abstract

**Background:**

Flexible denture base polymers have gained popularity in modern dentistry however, their biofilm formation tendency, adversely affecting the oral tissue heath, remains a concern. Consequently, this study aimed to evaluate surface roughness and biofilm formation tendency of two types of denture base resins manufactured with two techniques before and after surface coating with chlorohexidine (CHX) NPs.

**Materials and methods:**

Acetal (AC) and Polymethyl-methacrylate (PMMA) resins manufactured by conventional and CAD/CAM methods were shaped into disk (10 X 10 X 1 mm). They were dipped for 8 h and 24 h in colloidal suspension prepared by mixing aqueous solution of CHX digluconate and hexa-metaphosphate (0.01 M). Surface roughness, optical density (OD) of microbial growth media and biofilm formation tendency were evaluated directly after coating. Elutes concentrations of released CHX were evaluated for 19 days using spectrophotometer. Three-way ANOVA and *Tukey’s* post-hoc statistical analysis were used to assess the outcomes.

**Results:**

AC CAD/CAM groups showed statistically significant higher roughness before and after coating (54.703 ± 4.32 and 77.58 ± 6.07 nm, respectively). All groups showed significant reduction in OD and biofilm formation tendency after surface coating even after 19 days of CHX NPs release.

**Conclusions:**

Biofilm formation tendency was highly relevant to surface roughness of tested resins before coating. After CHX NPs coating all tested groups showed significant impact on microbial growth and reduction in biofilm formation tendency with no relation to surface roughness. Significant antimicrobial effect remained even after 19 days of NPs release and specimens storage.

## Background

Flexible dentures are excellent replacements to traditional methyl-methacrylate ones. They showed superior aesthetics, lighter weight, better adaptation to continual movement and flexibility especially in partially edentulous patients [1]. Polymer chemistry has provided dentistry with several of such materials like polyamides (nylon plastics), acetal resins (polyoxymethylene based materials), epoxy resins, polystyrene, polycarbonate resins, etc. [2].

Acetal (AC)-based dentures, which is one famous example of these materials, showed higher affinity to support candidal attachment than polyamide-based ones [3] and more micro-organisms were retained on mucosa under AC than metallic dentures [4]. That was attributed to its higher crystallinity and hence surface roughness. The latter could enhance more adhesion of oral micro-organisms and biofilm accumulation [5].

It was reported that inserting dentures within patient mouth leads to change within the ecology of the oral environment in addition to support of saprophytic environment formation. They both enhance certain oral micro-organisms growth such as *Candida albicans* [6, 7]*.* Furthermore, this encouragement for biofilm formation and adhesion to denture base could be one of the most reported factors for oral mucosa inflammation especially in poor oral hygiene or immune-deficiency patient and with high carbohydrates intake [8]. Thus improving self-cleaning or anti-microbial property of such dentures materials is of great importance.

Besides the material’s chemistry, the material’s manufacture technique is of great importance affecting final denture materials’ properties and performance intra-orally. Efforts were going to find a technique that could totally or partially reduce drawbacks of traditional denture fabrication technique which were used since several decades ago. The introduction of computer-aided designing/computer-aided manufacturing (CAD/CAM) technology is a good example of such techniques. Recent evidence based studies have demonstrated that CAD/CAM as a subtractive manufacture way provided poly-methyl-methacrylate (PMMA) based dentures with fracture toughness comparable to [9] or sometimes higher than PMMA manufactured with heat-polymerization technique [10, 11]. Consequently CAD/CAM dentures fabricated in easier and faster process could be a substituent to conventionally heat-polymerizable ones [9].

Several previous studies tried to improve the antimicrobial efficiency of denture materials either by incorporation of active antimicrobial elements such as phosphate groups into PMMA composition and reaction [12], introducing protein repellent like 2-methacryloyloxyethyl or silver nanoparticles (NPs) [13] or by developing surface film coating with antimicrobial prevalence [8]. A recent review article has concluded that colloidal silver in lower ratio could provide good antimicrobial effect to quaternary ammonium compounds when they are incorporated into denture resin [14]. They also reported that addition of 1 and 2% of graphene–Ag NPs to cold-cured denture resins showed decrease in cell viability of oral keratinocytes and dental pulp stem cells in addition to an antioxidant effect too [15].

Chlorhexidine (CHX) is a broad spectrum antibacterial and antifungal agent belonging to the biguanide class of drugs. One of its most common formulations used in dentistry is the water soluble digluconate form [16]. When it is combined with sodium hexametaphosphate they produce chlorhexidine–hexametaphosphate (CHX–HMP) colloidal suspension which previously proved to have antimicrobial efficiency in various dental fields such as surface implant coating [17], restorative materials [18] and denture silicon lining material [19], however, it was not used with denture base materials.

Nevertheless, CHX was utilized previously to inhibit candida biofilm formation onto PMMA denture surface through incorporating its diacetate salt, which is more ethanol than being water soluble, into polymerizing resin [20]. Although it showed a significant antifungal efficacy, it also significantly increased the water sorption of all tested denture base materials through increasing the micrometric pores within the material’s structure [20, 21]. In addition, studies comparing the surface properties of PMMA and AC resins with different manufacturing techniques are scarce especially for AC types and needs further focusing. Consequently, the current study aimed to enhance the antimicrobial ability of two denture base materials, AC-based polymers and PMMA, constructed by two different techniques, CAD/CAM and conventional methods, through dipping-method into a suspension of CHX–based nanoparticles (NPs). The null hypothesis assumed that:

1. There will be no difference in surface roughness (Ra) between tested materials before and after coating.
2. There will be no difference in biofilm formation tendency between different tested groups before and after NPs coating.
3. The coated groups will have no antimicrobial effect than non-coated group before and after evaluation of NPs release.

## Materials and methods

### Materials

Two types of denture base materials were used in this study AC-based polymer manufactured by CAD/CAM (Zirlux Acetal G2Bleach Henery Schein, Auckland, New Zealand) and flask injection method (Bioacetal™, Roko Dental Systems) and PMMA polymer manufactured by both CAD/CAM (HaHasmile pink PMMA blocks, Hahasnile®, Changash, China) and heat-cured compression molding (Vertex™ Implacryl, Vertex Dental, Netherland) all materials details are mentioned in Table 1.

**Table 1:** Materials used in the current study

Product	Manufacture	Processing technique
Vertex™ Implacryl	Vertex Dental, Netherland	Heat-cured PMMA
HaHasmile pink PMMA blocks	Hahasnile®, Changash, China.	CAD/CAM PMMA
Bioacetal™	Roko Dental Systems	Mold-injected poly-acetal
Zirlux Acetal G2Bleach	Henery Schein, Auckland, New Zealand.	CAD/CAM polyacetal
Chlorohexidine diglucontae, 20% w/v aq. Soln., non-sterile	Alfa Aesar, Cermany.	–
Sodium Hexametaphosphate (HMP)	Fisher Scientific, Acros Organics, Janssen Pharmaceutical, Belgium**.**	–

### Specimens preparation

Two hundreds and forty disk shaped specimens (10 X 10 X 1 mm) of the two tested materials were manufactured from both techniques (*n* = 10). For injection molding and compression molding techniques of both AC and acrylic resin, respectively, metal disks with the previous dimensions were applied to create the mold cavity space within the dental stone. For injecting AC specimens, prefabricated sprues were made by modeling wax into a specially designed flask for injecting the molten resin. Dewaxing followed to create sprue channels then applying a thin coat of a separating medium (acrylic-plaster separator, Alphabond Dental) to the created mold. Complete dryness was confirmed before additional processing. According to the manufacturer’s instructions, a cartridge of appropriate size was selected and placed in an electric cartridge furnace for softening of the AC resin at temperature of 250 °C and time period of 15–20 min. The molten resin was then injected into the mold cavities. This was performed using a digital molding machine (Multipress-digital molding machine-Roko). Finishing was performed to remove excess resin using finishing stone wheels and tungsten carbide burs to approve surface smoothness 150 grit size was used under copious amount of water cooling. Finally for surface finish, abrasive sandpaper papers were used with light manual pressure. For polishing, slurry of medium grit pumice mixed in a 1:1 ratio of water and two cloth wheels of 12.5 μm on the polishing lathe and polishing machine (Universal polishing; Ivoclar Vivadent) were applied in conjunction with a felt-polishing wheel (Shfuinnc, Kyoto) at a speed of 3000/rpm. Each cloth wheel was used for 60 sec. Acrylic specimens manufactured by compression molding technique followed the same previous finishing and polishing technique by the same operator [22].

For CAD/CAM specimens, rectangular specimens with the previously mentioned dimensions were milled. They were designed using software designer program (Exocad software, Dental DB 2.2 Valletta, Version 2.2 Engine build 6654) then milled using the former virtual design and computer supported milling machine (Ceramil motion 2, Amman Girrbach Austria) from both AC and PMMA blocks. Milled specimens received the previously mentioned finishing and polishing protocol from the same operator.

### Dipping into chlorohexidine-based nanoparticles (CHX NPs) suspension

First to prepare the CHX based NPs suspension, mixing of aqueous solutions of CHX digluconate (0.01 M) with sodium hexametaphosphate (HMP) (0.01 M) by volume percentage 1:1under continuous vigorous stirring (AREC F20500010, VELP Scientifica, Italy) at room temperature and pressure. Stirring was continued until a colloidal suspension with white precipitate was produced. The final suspension formed of 0.005 M concentration of both CHX and HMP. It was freshly prepared before specimens’ dipping coating by one hour. Specimens coating by dipping was done using the freshly prepared suspension for two periods 8 h (resembling the period where patient do not use denture throughout the day) and for longer period of 24 h.

Specimens were divided into twelve groups; six for AC: AC CAM as control (AC-CAM-C) without coating, AC-CAM dipped for 8 h and 24 h (AC-CAM-8 h and AC-CAM-24 h) similar three groups relative to AC injected to flask (AC-F) as AC-F-control (AC-F-C), AC-F-8 h and AC-F-24 h. The other six groups were relative to PMMA resin. Three for PMMA compression-molded into flask (PM-F) as control without coating PM-F-C, and two groups dipped for different periods as PM-F-8 h and PM-F-24 h. Three groups related to PMMA resin milled with computer aid as, PM-CAM-control (PM-CAM-C), and two groups dipped for different periods as PM-CAM-8 h and PM-CAM-24 h. Every coated group (*n* = 10) was dipped in 100 ml of the prepared suspension for the required period and under mechanical shaking using shaker (ATUART Flask shaker SF1, 230 V 50HZ, Bibby Scientific Limited, Staffordshire, UK) to ensure dispersion of white precipitate throughout the liquid. After dipping periods, all specimens were washed using distilled water for 5 sec and left in covered containers for 24 h until completely air dried before testing.

Afterwards, specimens were divided into two halves. Half of specimens were going through Ra, then optical density for microbial growth media and biofilm formation tendency. The other half was used to evaluate NPs release in distilled water for 19 days then for optical density of microbial growth media again.

### Atomic force microscope (AFM) for surface topography evaluation

Surface topography for all groups was conducted using scanning probe microscope (WET-SPM9600, Shimadzu, Japan) in non-contact mode. 120 specimens were used to evaluate surface roughness after surface coating for all groups. Measurement was conducted at room temperature and pressure. Three random measurement were conducted (5 X 5 μm in X and Y directions for each measurement) in the central part of each specimen, 2 mm away from each edge and the mean measurement for each specimen was taken. The expressed parameters for surface topography were Ra (the arithmetic average of absolute values of surface height deviations measured from the mean plane), Rq (the root-mean-square average of height deviations taken from the mean image data plane) and Rz (tallest “peak” and the deepest “valley” in the surface.), 2D and 3D images of the footprint areas.

### Biofilm assessment by crystal violet assay (CV) method

The crystal violet (CV) assay was used to test the ability of biofilm formation on different materials with slight modifications. The standard strains (*Pseudomonas aeruginosa* PAO1, *Staphylococcus aureus* ATCC6538, and *Candida albicans* ATCC 10231) were cultured on Muller Hinton broth (MHB) for *Pseudomonas aeruginosa* and *S. aureus* and MHB with 2% glucose for *C. albicans*, and incubated overnight at 37 °C. From overnight culture, a fresh aqueous suspension was prepared and adjusted to 0.5 McFarland turbidity then diluted 1/100 using fresh broth. The tested material disks were placed in different wells of 24-wells tissue culture plate then each well was filled with 1 mL of mixed inoculum suspension. Wells which contained only growth media were used as negative control and those with growth media with the tested micro-organisms without any material disks were used as positive control for media optical density (OD) test. Then the tissue culture plates were incubated for 48 h at 37 °C under stationary conditions. The well’s contents were aspirated and tested material disks were washed three times with phosphate buffered solution (PBS) then fixed for 20 min with 1 mL of 99% methanol, decanted, air dried, and stained for 15 min with 1 mL of 2% CV. Disks were left to dry then 500 μL of 33% glacial acetic acid was used to dissolve the bound stain [23]. OD of stained adherent biofilm was determined with ELISA reader at 630 nm wavelength**.** The ability of biofilm formation were classified into four groups based on the measured OD compared to OD of negative control as: non adherent (N), weakly (W) adherent, moderately (M) adherent, and strongly (S) adherent according to criteria of Stepanović et al. (2007) [24].

### Elution of soluble CHX from coated specimens

To detect the release of CHX in aqueous media for all groups after surface coating 120 specimens were stored in distilled water 3 ml/specimen in air tight closed containers. Measurements were conducted after first day, fourth, seventh, eleventh, fifteenth and nineteenth days. After the nineteenth day most groups started decrease in released amount so measurement stopped. After each of the former periods, specimens were placed in orbital shaker (150 rpm, SSM1; Bibby Scientific Limited, Staffordshire, UK) for two hours prior to elute valuation. They were then removed from their containers; the water amounts were renewed and specimens stored again in tightly closed containers. The elutes were collected to estimate the amount of CHX using transparent cuvettes. Unbounded CHX concentration were detected with absorbance mode at 225 nm [19] using spectrophotometer (C-7100, PEAK Instruments Inc., Houston, USA). CHX concentrations were calibrated with reference to standard solutions at 5–90 μM of CHX digluconate.

### Statistical analysis

The sample size calculation was done using the G* power analysis (v3.1.9.2, University of Dusseldorf) software. It showed minimal total number of specimen needed for each half of specimens = 112, one half for Ra, OD and biofilm and the other half for elutes evaluation and OD after release evaluation period. It was calculated at: effect size f = 0.403, % error probability 0.05, power of probability 85%, and degree of freedom (df) = 7. Statistical analysis was performed using SPSS 16.0 (SPSS, Chicago, IL, USA) for Windows. Results were first analyzed for normality using Shapiro Wilk followed by three-way ANOVA and *Tukey’s* post-hoc test for all tests; surface roughness (Ra), biofilm formation tendency, microbial growth and OD before and after 19 days of elutes release. *Student t-test* OD was conducted for OD before and after elutes release of CHX NPs.

## Results

### Surface roughness (Ra) by AFM

**Fig. 1:**
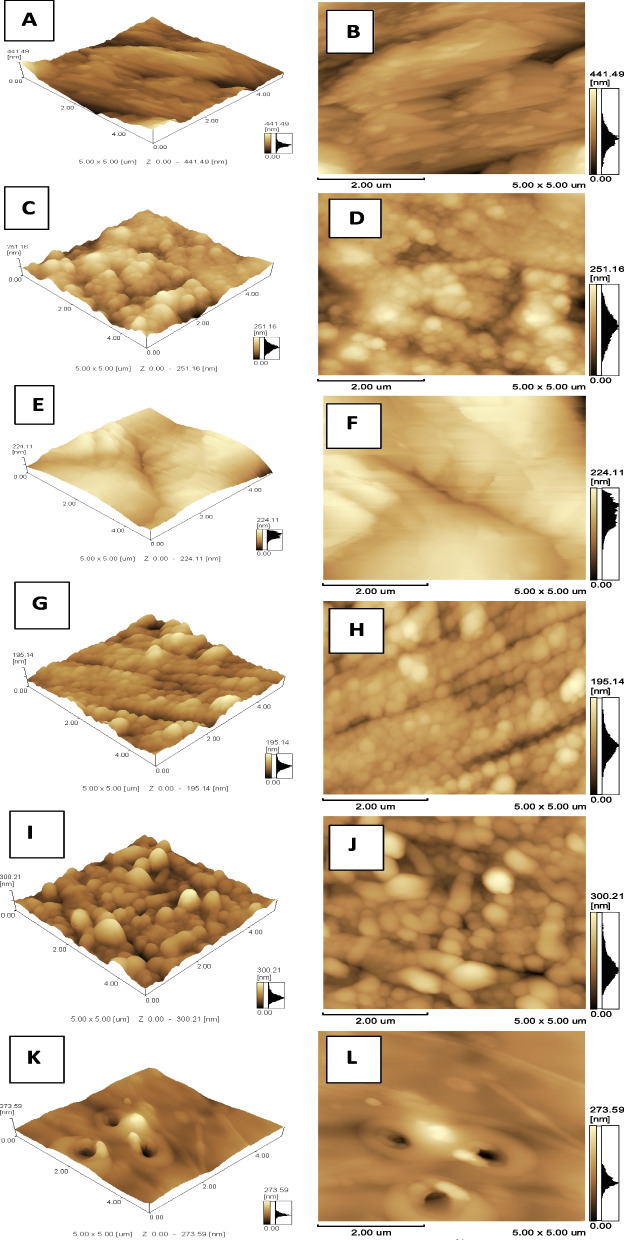
The AFM images (5 X 5 μm) for PMMA groups showing **A** & **B**, **C** & **D**, and **E** & **F** are 3D and 2D images for control, 8 h and 24 h of dipping in CHX NPs suspension, respectively, for PMMA with compression-molding technique. They show decrease in surface roughness (Ra) after both periods of dipping with retaining surface topography after 24 h dipping group. **G** & **H**, **I** & **J**, and **K** & **L** are 3D and 2D images for control, 8 h and 24 h of dipping in CHX NPs suspension, respectively, for PMMA with CAD/CAM technique. They show increase in Ra after both periods of dipping with retaining surface topography after 8 h than 24 h of dipping

**Fig. 2:**
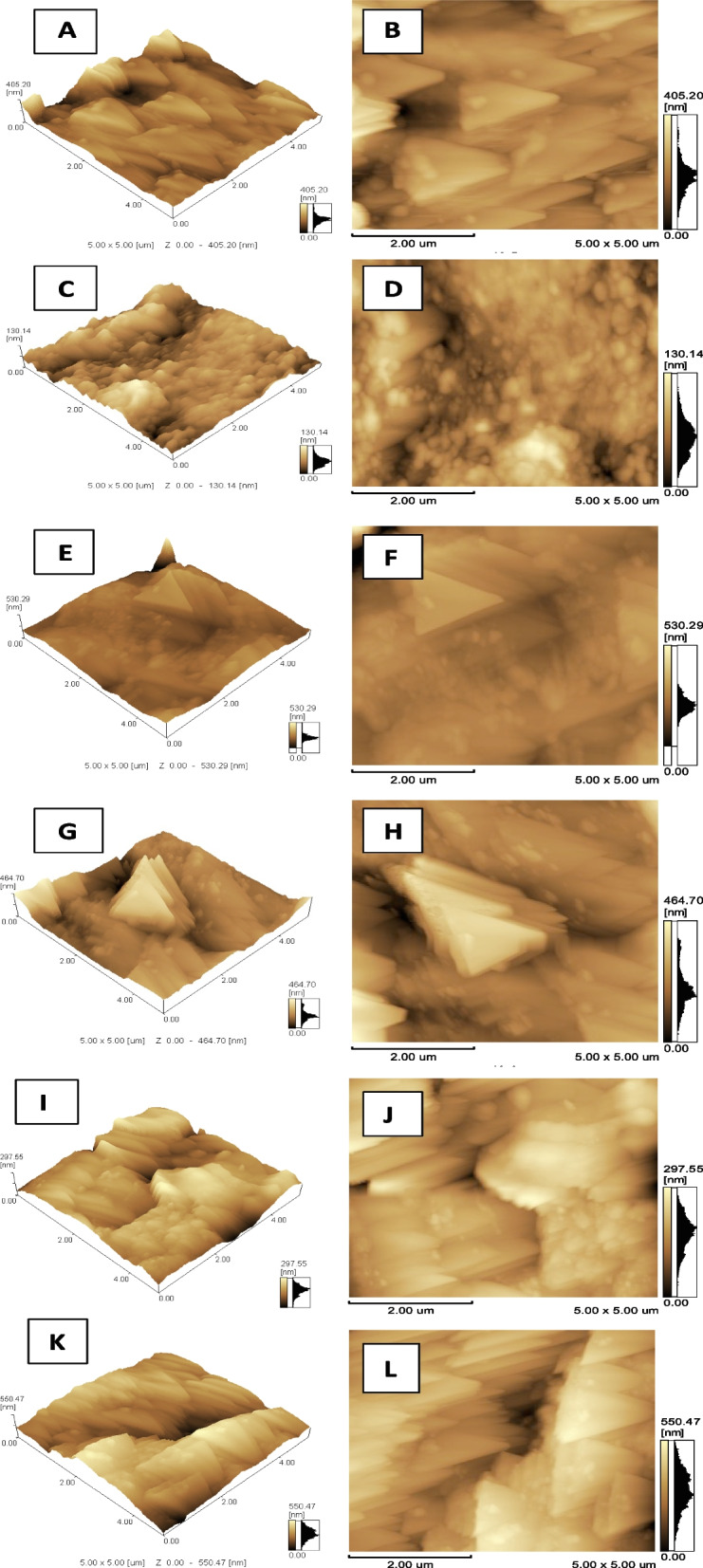
The AFM images (5 X 5 μm) for AC groups showing **A** & **B**, **C** & **D**, and **E** & **F** are 3D and 2D images for control, 8 h and 24 h of dipping in CHX NPs suspension, respectively, for AC with flask injection technique. They show much decrease in Ra after 8 h of dipping in comparison to control and 24 h of dipping with much higher Ra. The latter group retained the surface topography for the control group. **G** & **H**, **I** & **J**, and **K** & **L** are 3D and 2D images for control, 8 h and 24 h of dipping in CHX NPs suspension, respectively, for AC with CAD/CAM technique. They show similar Ra results for the flask injection types. The surface topography is almost the same in all cases for CAD/CAM groups

Three-way ANOVA analysis showed statistical significant impact (*p-value* = 0.01) of the three variables (materials, manufacture technique and time) and all possible interaction between them on Ra (Table 2). Generally within coated groups the AC-CAD-24 h coated for 24 h showed the highest statistically significant (*p-value* = 0.00) Ra among all groups followed by its control group. On the other hand, the relevant group coated for 8 h showed much lower Ra that is comparable to the control groups manufactured with conventional methods (PM-F-C and AC-F-C). AC-F-8 h group coated for 8 h showed the highest decrease in Ra than its control and the significantly lowest Ra among all groups (Table 6). Figures 1 and 2 show the surface topography of all groups and grain size that is concurrent with the increase or decrease in Ra.

**Table 2:** Three-way ANOVA showing factorial interaction between variables impact on surface roughness (Ra)

Source	Type III Sum of Squares	df	Mean Square	F	Sig.
Corrected Model	9899.664	11	899.969	48.535	.000
Intercept	43,809.281	1	43,809.281	2.363E3	.000
Material	2448.270	1	2448.270	132.035	.000
Manufcature technique	657.068	1	657.068	35.435	.000
Time	963.750	2	481.875	25.987	.000
Material X Manufcature	2625.538	1	2625.538	141.595	.000
Material X Time	2194.173	2	1097.087	59.166	.000
Manufcature X Time	631.076	2	315.538	17.017	.000
Material X Manufcature X Time	379.790	2	189.895	10.241	.001

Table 5 shows the impact of all variables on biofilm formation tendency after coating. The biofilm formation tendency was the highest for AC-CAD-C group that showed the highest Ra among control groups. However, the treated AC-CAD-8 h and AC-CAD-24 h groups showed statistically significant (*p-value* = 0.00) decrease in biofilm tendency formation even though the AC-CAD groups had the significantly highest Ra among all groups. The latter showed comparable biofilm formation tendency to control groups PM-F-C and PM-CAD-C. Other treated groups showed no growth in comparison with negative control (growth media with no micro-organisms) (Table 6).

### Optical density (OD) of microbial growth media before & after CHX elutes evaluation and tendency for biofilm formation

**Fig. 3:**
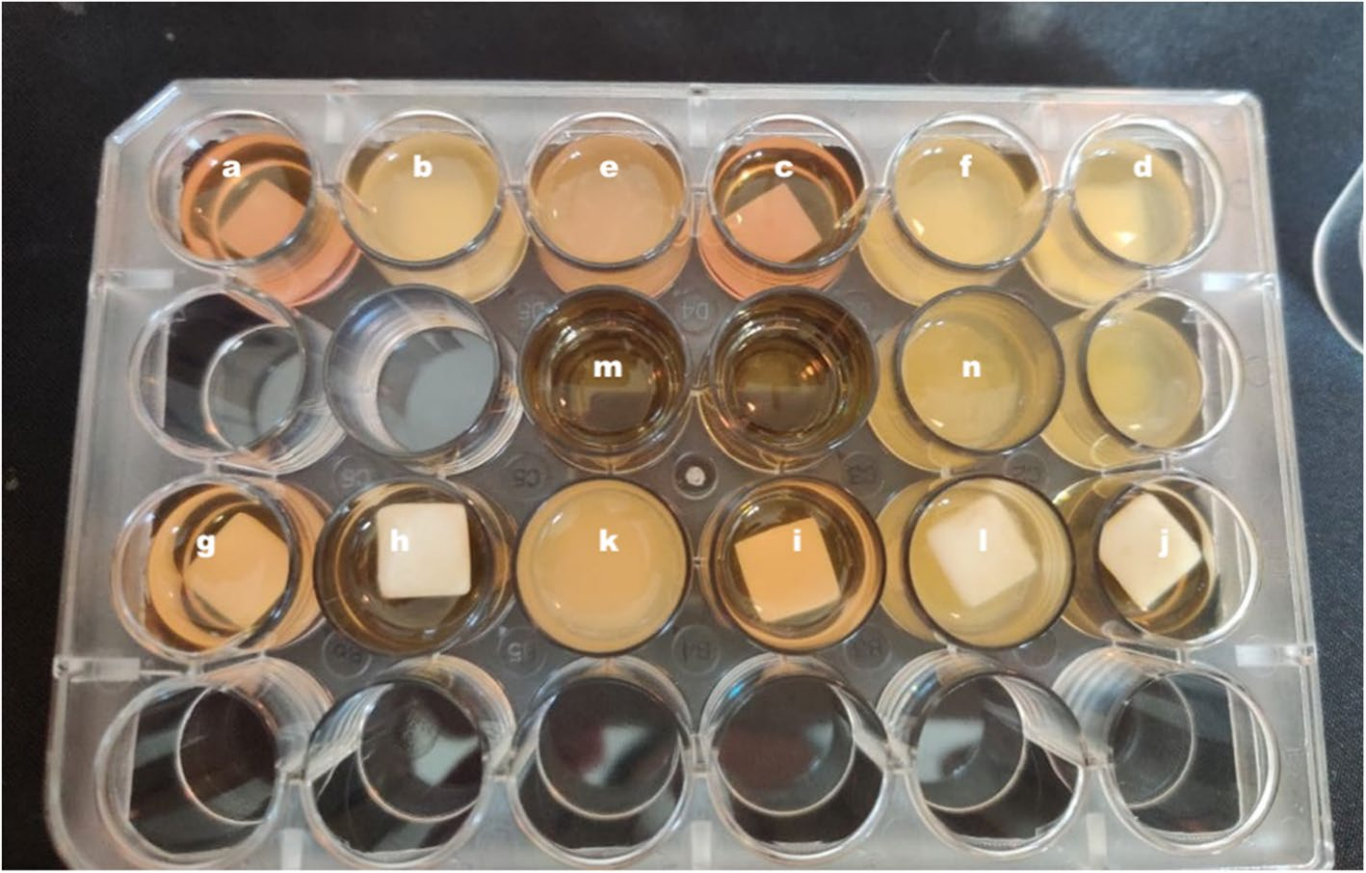
This figure shows turbidity of the microbial growth media obtained for all groups directly after dipping coating procedures. a & b and c & d are PM-CAD-8 h & AC-CAD-8 h and PM-CAD-24 h & AC-CAD-24 h, respectively, e & f are PM-CAD and AC-CAD controls, respectively. g & h and i & j are PM-F-8 h & AC-F-8 h and PM-F-24 h & AC-F-24 h, respectively, k and l are for PM-F and AC-F controls. The photo shows highest turbidity for all control groups whereas AC-CAD 8 showed the highest turbidity among treated groups. Finally, m & n are for negative and positive controls, respectively

**Fig. 4:**
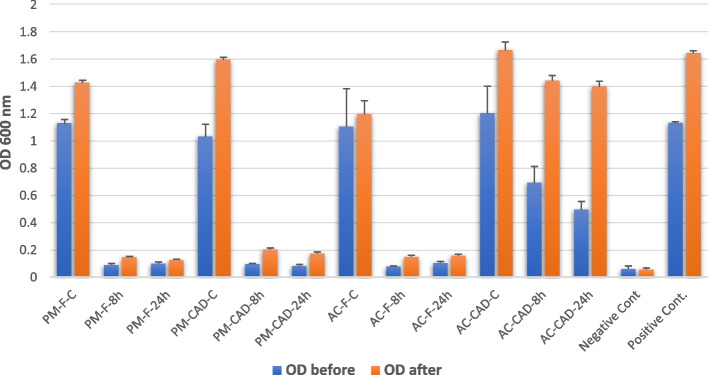
Bar-chart showing the optical density (OD) denoting microbial growth for all groups before and after storage and release of CHX-based NPs

Three-way ANOVA analysis showed a statistically significant impact (*p-value* = 0.00) on OD before CHX elutes evaluation and after storage in distilled water for 19 days. However, after elutes evaluation, all variables and interactions between them showed high impact on OD. However, interaction between composition & time and between three variables showed no significant impact (*p-value* = 0.069 and 0.096) (Tables 3 and 4). *Student t-test* showed statistically significant decrease in OD (*p-value* = 0.007) after 19 days of storage and release (Table 6 and Figs. 3 and 4).

**Table 3:** Three-way ANOVA showing factorial interaction between variables impact on growth media OD (before CHX release evaluation)

Source	Type III Sum of Squares	df	Mean Square	F	Sig.
Corrected Model	7.694	11	.699	56.396	.000
Intercept	9.642	1	9.642	777.427	.000
Composition	.329	1	.329	26.488	.000
Technique	.249	1	.249	20.090	.000
Time	6.469	2	3.235	260.799	.000
Composition X Technique	.364	1	.364	29.334	.000
Composition X Time	.074	2	.037	2.999	.069
Technique X Time	.145	2	.072	5.835	.009
Composition X Technique X Time	.064	2	.032	2.588	.096

**Table 4:** Three-way ANOVA showing factorial interaction between variables impact on growth media OD (after CHX release evaluation)

Source	Type III Sum of Squares	df	Mean Square	F	Sig.
Corrected Model	15.464	11	1.406	934.184	.000
Intercept	23.468	1	23.468	1.559E4	.000
Composition	1.363	1	1.363	906.011	.000
Technique	2.674	1	2.674	1.777E3	.000
Time	7.925	2	3.963	2.633E3	.000
Composition X Technique	1.862	1	1.862	1.238E3	.000
Composition X Time	.989	2	.495	328.741	.000
Technique X Time	.231	2	.116	76.859	.000
Composition X Technique X Time	.418	2	.209	138.885	.000

**Table 5:** Three-way ANOVA showing factorial interaction between variables impact on biofilm formation directly after specimens coating

Source	Type III Sum of Squares	df	Mean Square	F	Sig.
Corrected Model	.199	11	.018	684.441	.000
Intercept	1.219	1	1.219	4.610E4	.000
Composition	.103	1	.103	3.901E3	.000
Technique	.035	1	.035	1.329E3	.000
Time	.008	2	.004	147.827	.000
Composition X Technique	.052	1	.052	1.963E3	.000
Composition X Time	.001	2	.000	16.009	.000
Technique X Time	6.650E-5	2	3.325E-5	1.257	.302
Composition X Technique X Time	.000	2	7.619E-5	2.881	.076

**Table 6:** Means ± Standard deviations (SD) for Ra, growth media OD before and after CHX release evaluation & biofilm formation tendency and *student t-test* for OD before and after CHX release

Groups	Mean ± SD Ra (nm)	Mean ± SD OD before	Mean ± SD OD after	*Student t-test* for OD	Mean ± SD Biofilm formation tendency
PM-F-C	38.38 ± 3.84 ^**c**^	1.128 ± 0.03 ^**a**^	1.426 ± 0.02 ^**b**^	**t**_**(11) = 3.340**_ **&** ***p-value =*** 0.007^*^	1.202 ± 0.086 ^**c**^
PM-F-8 h	28.63 ± 3.28 ^**cde**^	0.090 ± 0.01 ^**c**^	0.148 ± 0.004 ^**de**^		NG
PM-F-24 h	25.70 ± 4.95 ^**def**^	0.101 ± 0.01 ^**c**^	0.128 ± 0.003 ^**de**^		NG
PM-CAD-C	18.37 ± 3.91 ^**ef**^	1.033 ± 0.09 ^**a**^	1.595 ± 0.02 ^**a**^		1.265 ± 0.053 ^**bc**^
PM-CAD-8 h	30.31 ± 5.14 ^**cde**^	0.097 ± 0.003 ^**c**^	0.204 ± 0.01^**d**^		NG
PM-CAD-24 h	18.42 ± 3.42 ^**ef**^	0.084 ± 0.01 ^**c**^	0.175 ± 0.01 ^**d**^		NG
AC-F-C	39.89 ± 4.40 ^**c**^	1.104 ± 0.28 ^**a**^	1.196 ± 0.10 ^**c**^		1.459 ± 0.008 ^**b**^
AC-F-8 h	15.943 ± 4.13 ^**f**^	0.080 ± 0.002 ^**c**^	0.151 ± 0.01 ^**de**^		NG
AC-F-24 h	35.12 ± 3.87 ^**cd**^	0.105 ± 0.01 ^**c**^	0.159 ± 0.01 ^**de**^		NG
AC-CAD-C	54.703 ± 4.32 ^**b**^	1.202 ± 0.20 ^**a**^	1.666 ± 0.06 ^**a**^		1.700 ± 0.171 ^**a**^
AC-CAD-8 h	35.55 ± 3.52 ^**cd**^	0.693 ± 0.12 ^**b**^	1.441 ± 0.04 ^**b**^		1.266 ± 0.021 ^**bc**^
AC-CAD-24 h	77.58 ± 6.07 ^**a**^	0.496 ± 0.06 ^**b**^	1.399 ± 0.04 ^**b**^		1.305 ± 0.001 ^**bc**^
Negative Cont	–	0.062 ± 0.02 ^**c**^	0.058 ± 0.01 ^**e**^		–
Positive Cont.	–	1.131 ± 0.01^**a**^	1.643 ± 0.02 ^**a**^		–

Letters are for *Tukey’s* test for each test individually. ^*^ means significant difference for *p*-value. NG means no microbial growth was observed for these groups

**Fig. 5:**
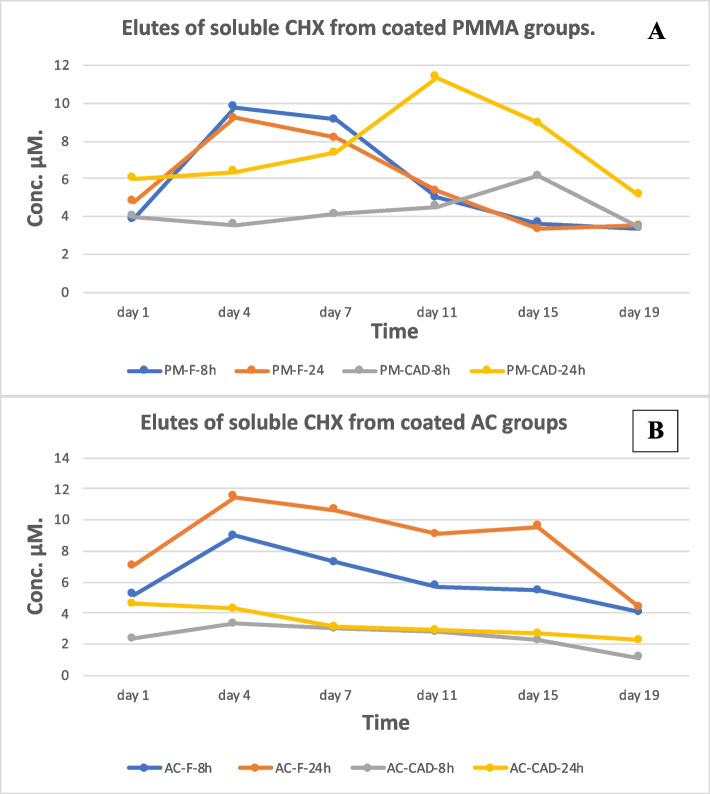
The soluble CHX release mode (μM) in both types of surface treated denture base resins (**A** for PMMA and **B** for AC groups). They show higher release in groups treated for 24 h than treated for 8 h in most cases. Release was mostly higher middle region of the curve except for AC-CAD-24 h

For soluble CHX elutes evaluation, all groups showed almost similar pattern where maximum CHX release concentration values were mostly in the middle region. All PMMA groups started with close values for release (Fig. 5A) while there was wider range of release concentrations values in the first day for AC groups (Fig. 5B). All groups treated with 24 h showed higher release than groups treated for 8 h (Fig. 5). The data base used and/or analyzed during the current available from correspondent author on researchable request.

## Discussion

This study focused on evaluating two types and techniques of denture base construction and their antimicrobial ability after coating their surface by CHX NPs. Previous reporting showed evidence of remaining deposits of CHX NPs on denture lining materials surface after 112 days of immersion in artificial saliva in particular CHX NPs prepared with HMP [19]. CHX NPs prepared through formation of colloidal suspension through reacting with hexa-meta phosphate (HMP) was prepared and characterized previously. They reported formation of highly negatively charged NPs with quick adherence to titanium, glass and rubber materials [25].

The current study null hypothesis suggested that no difference between Ra and antimicrobial ability of tested materials prepared by two different techniques neither their surface coating with CHX NPs would have an effect too. Firstly, findings of the current study proved rejection of the first assumption as there was significant difference within Ra values among all control groups. Generally, the current Ra values were lower than relative studies [26, 27]. Although, Ra results for conventional PMMA are in accordance with reported range for PMMA resin (0.03 and 0.75 μm) [28] current Ra results for PMMA with CAD/CAM technique still showing lower values. That could be explained by the method used to evaluate the Ra using AFM in non-contact mode where previous studies used profilometer. Furthermore, Ra is dependent on other variables like properties of each used materials, polishing technique and manual skills of the operator [27], all polishing procedures were conducted with single operator in the current study. Within PMMA control groups the Ra was much higher for compression molding technique than CAD/CAM group. This is in agreement with previous work showing that Ra of conventional PMMA was almost double that of CAD/CAM PMMA [29]. This finding could be attributed to manufacture process of machinable PMMA blocks. Their powder and the liquid of proper proportions are mixed and its dough is stored at a low temperature (− 18 °C) for about 24 h. Afterwards, it is placed into molds, pressed then gradually polymerized with slow increase in temperature. The target of such whole processing technique is to enhance the degree of polymerization without creating voids within the blocks. This results in forming a resin with very long polymer chains, low residual monomer content and elevated hardness [30, 31].

Reports regarding Ra of AC resins, prepared either with injection or CAD/CAM methods, are still too scarce. However, current findings showed higher Ra for control uncoated AC groups (AC-F-C and AC-CAD-C) than PMMA (PM-F-C and PM-CAD-C) groups with no significant difference between PMMA prepared with conventional method and AC with CAD/CAM technique. These results are in agreement with previous study [26] where AC showed higher Ra than PMMA where both were prepared by injection molding system. This could be attributed to high degree of crystallinity reported for polyoxymethlene (AC resins). It is reported to be a semi-crystalline, thermoplastic polymer with high degree of crystallinity [32]. There was a general agreement concerning the role played by high surface roughness in enhancement of initial bacterial and/or fungal adherence and colonization onto dental biomaterials’ surface [32]. The current results of uncoated groups put more emphasis on the previous findings where AC-CAD-C and AC-F-C with the significantly highest Ra showed the highest biofilm formation tendency.

After surface coating with CHX NPs, there were large variations within treated and untreated groups in Ra with the highest Ra for AC-CAD-24 h. However, all groups showed less Ra values than the reported threshold level (0.2 μm) below which no more bacterial retention to surface could occur [28]. There was previous trials to improve the antimicrobial efficacy of PMMA denture base materials using NPs like titanium dioxide and silver NPs [8, 13]. Most trials incorporate NPs as fillers with different percentages which needs adequate distribution and negatively affected tensile strength [13]. CHX was also incorporated into PMMA dentures as CHX diacetate salt. It proved to have significant effect on *candida albicans* growth however it increased PMMA water sorption in added concentrations of 1 and 2 wt% [20]. The current study utilized an easy method for NPs application by dipping method for 8 h, resembling the period where patient removes the denture per day, and for longer period 24 h to study more exposure effect. The former method is considered a simple approach that could be performed by the patient himself. It avoids the liability of affecting the denture base polymerization and or uneven distribution of added particles. Additionally, it is easier to be used with dentures already produced from machinable milling process or resins prepared with injection molding at high temperature like polyoxymethylene resins.

The antimicrobial efficacy of surface treated materials showed significant impact in growth media OD. That indicates inhibition of *C. albicans*, *S. aureus* and *Pseudomonas aeruginosa* growth within the growth media. These findings agree with previous work where incorporated CHX diacetate created significant inhibition impact when added to PMMA by 0.5 to 2 wt% [20]. CHX NPs prepared through reaction with HMP proved to significantly inhibit *C. albicans* growth on silicon denture base lining materials [19]. Furthermore, ethylene vinyl acetate polymers, used for many biomedical uses like mouth guards, when immersed in colloidal suspension of CHX and HMP released amount of CHX NPs that was capable of significantly inhibit the growth of *S. aureus* and *Pseudomonas aeruginosa* [33]*.* The latter results are in accordance with the current findings where all treated groups showed significant impact on microbial growth media OD after coating and after a period of 19 days of storage and CHX NPs elutes evaluation.

Among the treated groups, AC-CAD (8 h and 24 h) showed the highest OD of growth media and least tendency for biofilm inhibition efficacy in comparison with other treated groups. POM is known as thermoplastic polymer with a monomer-free crystalline structure consisting of a chain of alternating methyl groups linked by an oxygen molecule. It is produced by the polymerization of formaldehyde [26, 34]. In addition, it s a hydrophobic material meaning that it has low tendency for water or saliva absorption with little or no porosity [26]. Regarding the fact that AC-CAD blocks are manufactured under more controlled conditions of temperature and pressure, similar to the case of PMMA machinable blocks [30, 31], which may result in higher degree of crystallinity, minimal porosity and water sorption than the flask injected type. Current OD results and biofilm formation tendency are in accordance with the results of CHX NPs release in elutes from coated groups. Results of AC-ACD treated groups showed lowest amount of release among all treated groups.

Properties of PMMA polymers are dependent on their microstructure as they are highly complex macromolecules with heterogenous conformation having un-continuous empty spaces and unequal interstices [35]. Polar nature of such polymer, the former spaces [36] in addition to amount of residual monomer are responsible for water sorption [37]. This is in contrast to POM which is monomer-free and has minimal porosity [26, 29]. The current findings need to be more verified through in vivo studies simulating behavior of these coated resins in patients mouth harsh oral environment of fluctuating pH and variable patients’ habits. In addition to the previous limitation of the current study, evaluation of CHX NPs release is to be done for longer periods than currently done. However, evaluation of CHX NPs release was stopped directly after its onset of decline in most groups. That was done to reevaluate the efficacy of such released amount on inhibiting the biofilm micro-organisms in growth media. Although the OD values were significantly decreased after the period of 19 days yet treated groups were still showing significant decrease in OD of microbial growth media. Further researches are needed to evaluate other surface properties like micro-hardness, wettability and color change.

## Conclusions

Within the limitations and based on the findings of the current study, AC and PMMA manufactured with both conventional and CAD/CAM approaches for denture base showed difference in their final surface roughness that is directly related to their tendency to biofilm formation. AC-CAD showed the highest roughness. Treating the former resins with CHX NPs colloidal suspensions significantly improved their anti-microbial and biofilm formation tendency irrelevant to surface roughness after coating. Significant antimicrobial effect remained even after 19 days of NPs release and specimens storage.

## Data Availability

The data base used and/or analyzed during the current available from correspondent author on researchable request.

## References

[1] Singh J, Dhiman R, Bedi RPS, Girish S (2011). Flexible denture base material: a viable alternative to conventional acrylic denture base material. Contemp Clin Dent..

[2] Ardelean L, Bortun CM, Podariu AC, Rusu LC, Das CK (2015). Thermoplastic resins used in dentistry. Thermoplastic Elastomers. Synthesis and Applications.

[3] De Freitas Fernandes FS, Pereira-Cenci T, Da Silva WJ, Filho APR, Straioto FG, Del Bel Cury AA (2011). Efficacy of denture cleansers on Candida spp. biofilm formed on polyamide and polymethyl methacrylate resins. J Prosthet Dent..

[4] Al-Akhali M, El-Kerdawy M, Ibraheim Z, Abbas N (2012). Comparative study on the microbial adhesion to acetal resin and metallic removable partial denture. Indian J Dent..

[5] Bahrani F, Vojdani M, Safari A, Karampoor G (2012). Comparison of hardness and surface roughness of two denture bases polymerized by different methods. World J Dent..

[6] Martins N, Ferreira ICFR, Barros L, Silva S, Henriques M (2014). Candidiasis: predisposing factors, prevention, diagnosis and alternative treatment. Mycopathologia..

[7] Garcia-Cuesta C, Mg S-P, Jv B. Current treatment of oral candidiasis: a literature review. J Clin Exp Dent. 2014:e576–82. 10.4317/jced.51798. 10.4317/jced.51798 PMC4312689 25674329

[8] Géczi Z, Hermann P, Kőhidai L, Láng O, Kőhidai Z, Mészáros T (2018). Antimicrobial silver-Polyethyleneimine-Polylactic acid polymer composite film for coating methacrylate-based denture surfaces. J Nanomater..

[9] Abualsaud R, Gad M (2022). Flexural strength of CAD/CAM denture base materials: systematic review and meta-analysis of in-vitro studies. J Int Soc Prev Community Dent..

[10] Hada T, Kanazawa M, Iwaki M, Katheng A, Minakuchi S (2021). Comparison of mechanical properties of PMMA disks for digitally designed dentures. Polymers..

[11] Aguirre BC, Chen J-H, Kontogiorgos ED, Murchison DF, Nagy WW (2020). Flexural strength of denture base acrylic resins processed by conventional and CAD-CAM methods. J Prosthet Dent..

[12] Raj PA, dentino andrew. New phosphated poly(methyl methacrylate) polymers for the prevention of denture-induced microbial infection: an in vitro study. Clin Cosmet Investig. Dent. 2011:25. 10.2147/CCIDEN.S16860. 10.2147/CCIDEN.S16860 PMC3652354 23674911

[13] Bangera MK, Kotian R, Madhyastha P (2023). Effects of silver nanoparticle-based antimicrobial formulations on the properties of denture polymer: a systematic review and meta-analysis of in vitro studies. J Prosthet Dent..

[14] Cervino G, Cicciù M, Herford AS, Germanà A, Fiorillo L (2020). Biological and chemo-physical features of denture resins. Materials..

[15] Bacali C, Baldea I, Moldovan M, Carpa R, Olteanu DE, Filip GA (2020). Flexural strength, biocompatibility, and antimicrobial activity of a polymethyl methacrylate denture resin enhanced with graphene and silver nanoparticles. Clin Oral Investig..

[16] Ryalat S, Darwish R, Amin. New form of administering chlorhexidine for treatment of denture-induced stomatitis. Ther Clin Risk Manag. 2011:219–25. 10.2147/TCRM.S18297. 10.2147/TCRM.S18297 PMC3132092 21753884

[17] Wood NJ, Jenkinson HF, Davis SA, Mann S, O’Sullivan DJ, Barbour ME (2015). Chlorhexidine hexametaphosphate nanoparticles as a novel antimicrobial coating for dental implants. J Mater Sci Mater Med..

[18] da Silva MER, Danelon M, Santos Souza JA, Silva DF, Pereira JA, Pedrini D (2019). Incorporation of chlorhexidine and nano-sized sodium trimetaphosphate into a glass-ionomer cement: effect on mechanical and microbiological properties and inhibition of enamel demineralization. J Dent..

[19] Garner SJ, Nobbs AH, McNally LM, Barbour ME (2015). An antifungal coating for dental silicones composed of chlorhexidine nanoparticles. J Dent..

[20] Maluf CV, Peroni LV, Menezes LR, Coutinho W, Lourenço EJV, Telles D de M. Evaluation of the physical and antifungal effects of chlorhexidine diacetate incorporated into polymethyl methacrylate. J Appl Oral Sci. 2020;28:e20190039. 10.1590/1678-7757-2019-0039. 10.1590/1678-7757-2019-0039 PMC6919199 31939520

[21] Bayraktar G, Guvener B, Bural C, Uresin Y (2006). Influence of polymerization method, curing process, and length of time of storage in water on the residual methyl methacrylate content in dental acrylic resins. J Biomed Mater Res B Appl Biomater..

[22] Fathy SM, Emera RMK, Abdallah RM (2021). Surface microhardness, flexural strength, and clasp retention and deformation of Acetal vs poly-ether-ether ketone after combined thermal cycling and pH aging. J Contemp Dent Pract..

[23] El-Ganiny AM, Shaker GH, Aboelazm AA, El-Dash HA (2017). Prevention of bacterial biofilm formation on soft contact lenses using natural compounds. J Ophthalmic Inflamm Infect..

[24] Stepanović S, Vuković D, Hola V, Bonaventura GD, Djukić S, Ćirković I (2007). Quantification of biofilm in microtiter plates: overview of testing conditions and practical recommendations for assessment of biofilm production by staphylococci. APMIS..

[25] Barbour M, Maddocks S, Wood N, Collins A. Synthesis, characterization, and efficacy of antimicrobial chlorhexidine hexametaphosphate nanoparticles for applications in biomedical materials and consumer products. Int J Nanomedicine. 2013:3507–19. 10.2147/IJN.S50140. 10.2147/IJN.S50140 PMC3787925 24092973

[26] Mekkawy MA, Hussein LA, Alsharawy MA (2015). Comparative study of surface roughness between polyamide, thermoplastic polymethyl methacrylate and acetal resins flexible denture base materials before and after polishing. Life Sci J..

[27] Al-Dwairi ZN, Tahboub KY, Baba NZ, Goodacre CJ, Özcan M (2019). A comparison of the surface properties of CAD/CAM and Conventional Polymethylmethacrylate (PMMA). J Prosthodont..

[28] Alp G, Johnston WM, Yilmaz B (2019). Optical properties and surface roughness of prepolymerized poly(methyl methacrylate) denture base materials. J Prosthet Dent..

[29] Al-Fouzan AF, Al-mejrad LA, Albarrag AM (2017). Adherence of *Candida* to complete denture surfaces *in vitro* : a comparison of conventional and CAD/CAM complete dentures. J Adv Prosthodont..

[30] WO2018009518A1-Multiple layered denture block and/or disk, 2018. https://patents.google.com/patent/WO2018009518A1/en

[31] A61C13/0004 - Computer-assisted sizing or machining of dental prosthese. Cad/cam-machinable disc for the manufacture of fiber inlay-cores, 2017. https://patents.google.com/patent/CA3007885A1/en

[32] El-Din SM, Badr AM, Agamy EM, Mohamed GF (2018). Effect of two polishing techniques on surface roughness of three different denture base materials (an in vitro study). Alex Dent J..

[33] Barbour M, Wood N, Maddocks S, Grady H, Collins A. Functionalization of ethylene vinyl acetate with antimicrobial chlorhexidine hexametaphosphate nanoparticles. Int J Nanomedicine. 2014:4145. 10.2147/IJN.S65343. 10.2147/IJN.S65343 PMC4157624 25206305

[34] Tannous F, Steiner M, Shahin R, Kern M (2012). Retentive forces and fatigue resistance of thermoplastic resin clasps. Dent Mater..

[35] Miettinen VM, Vallittu PK (1997). Water sorption and solubility of glass fiber-reinforced denture polymethyl methacrylate resin. J Prosthet Dent..

[36] Braden M (1964). The absorption of water by acrylic resins and other materials. J Prosthet Dent..

[37] Arikan A, Ozkan YK, Arda T, Akalin B (2010). Effect of 180 days of water storage on the transverse strength of acetal resin denture base material. J Prosthodont..

